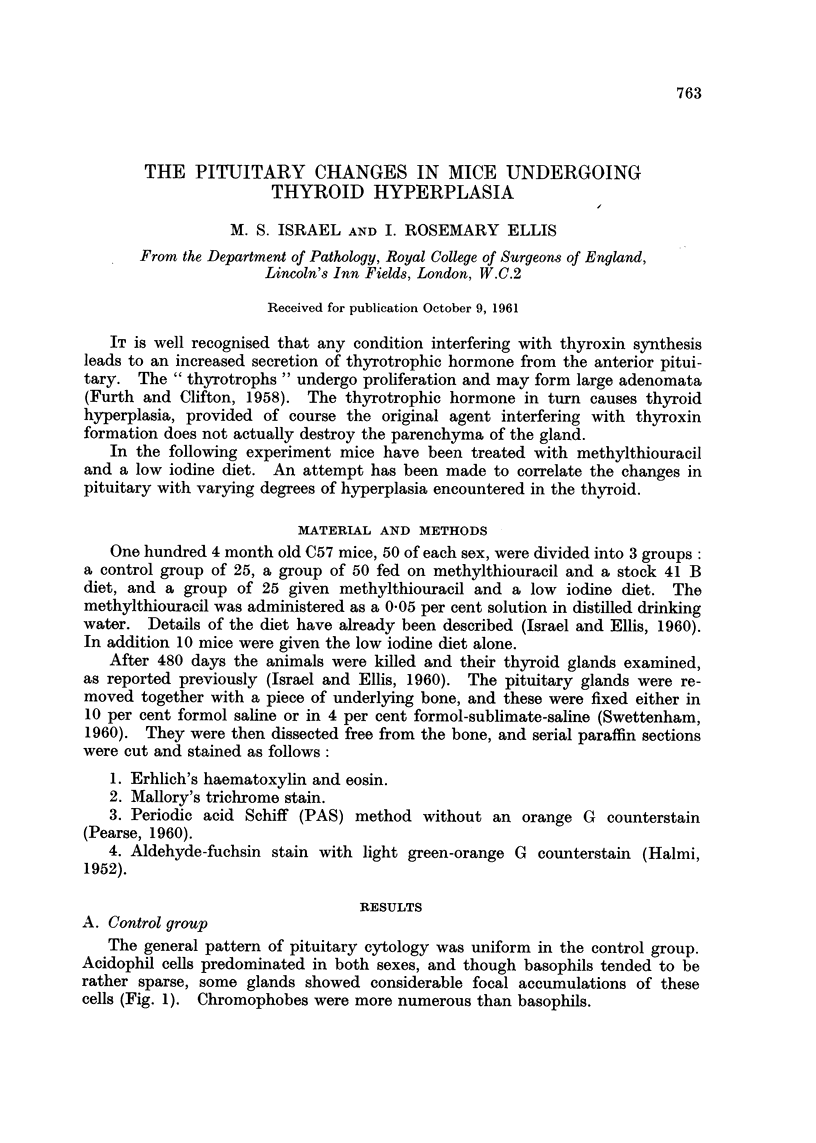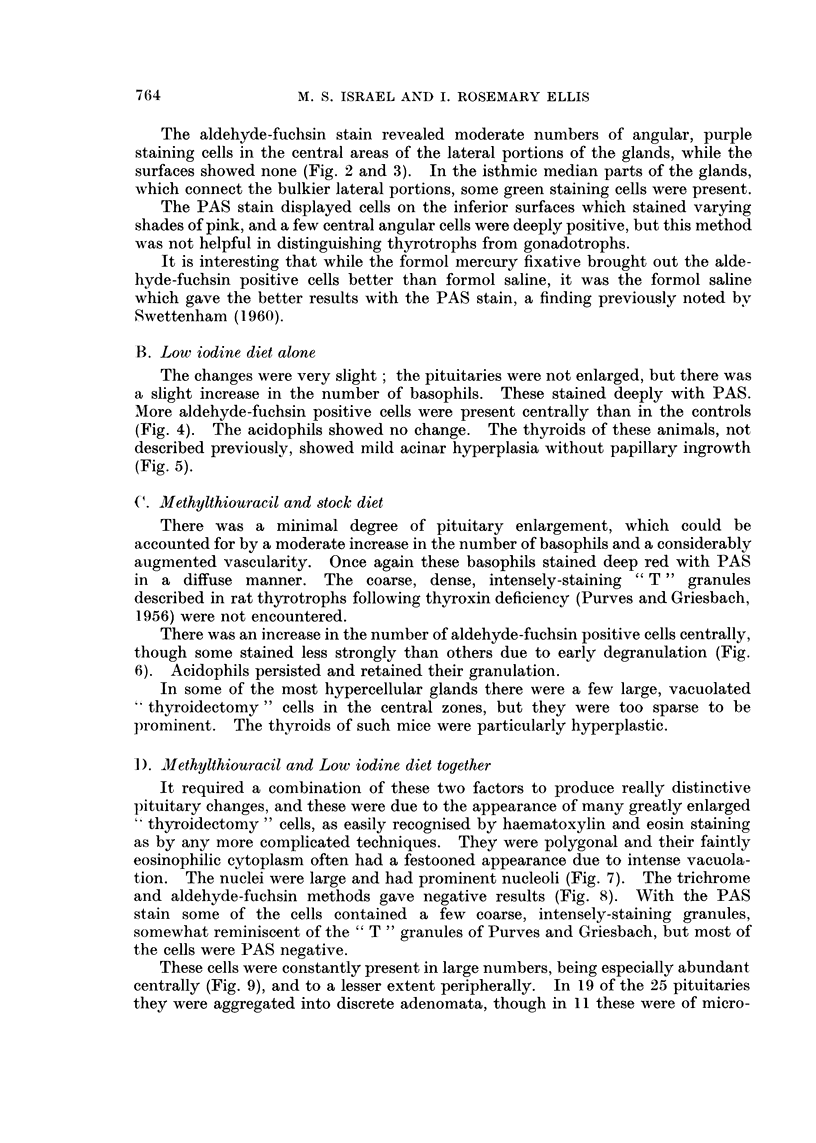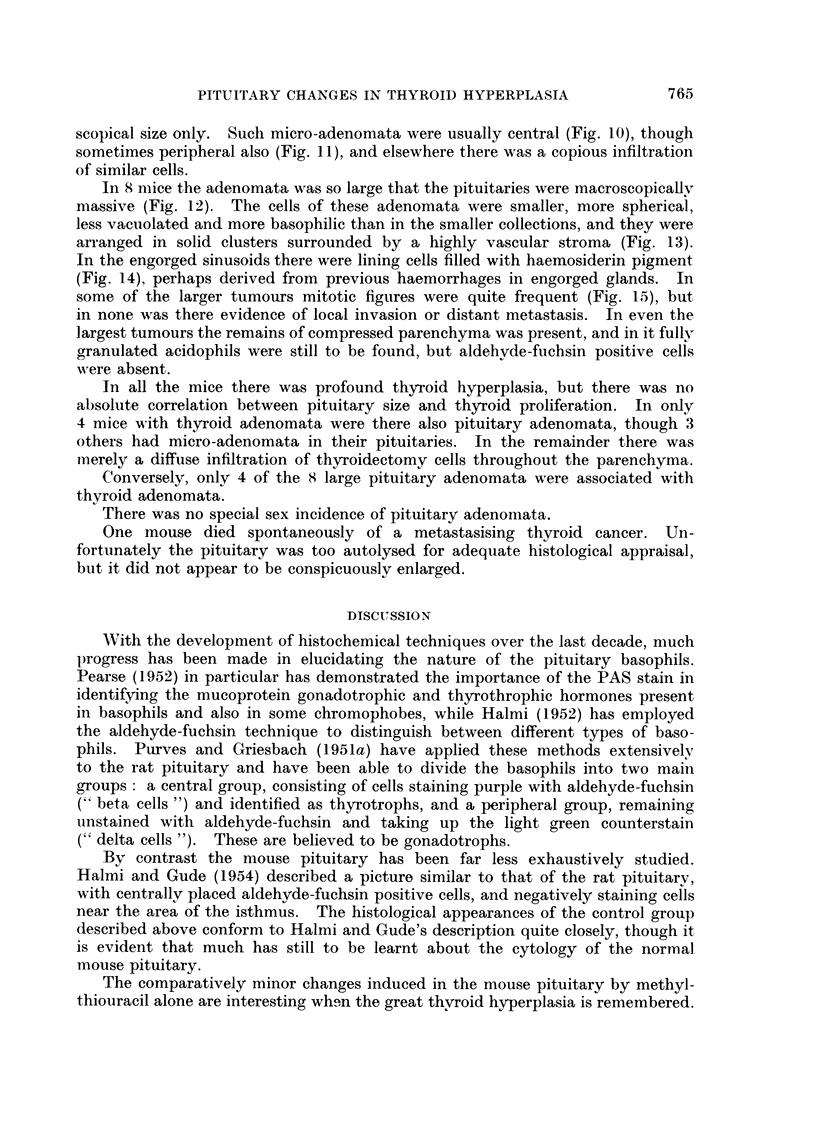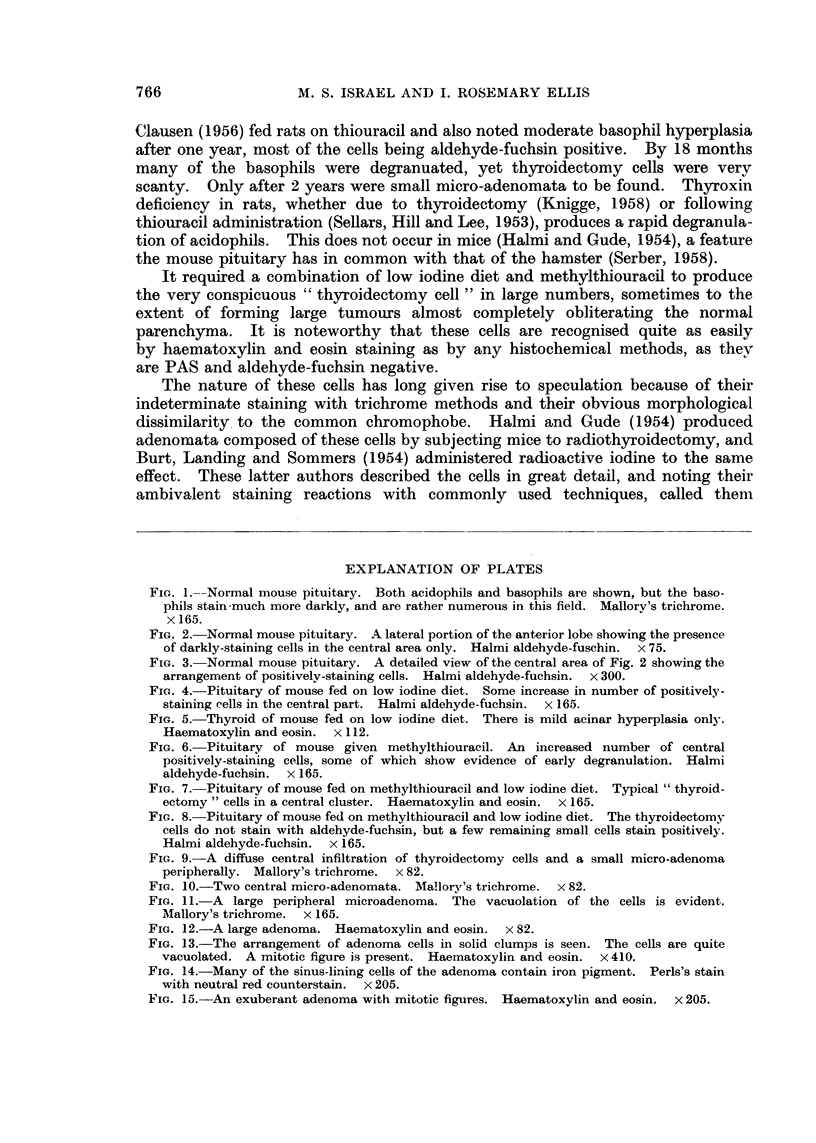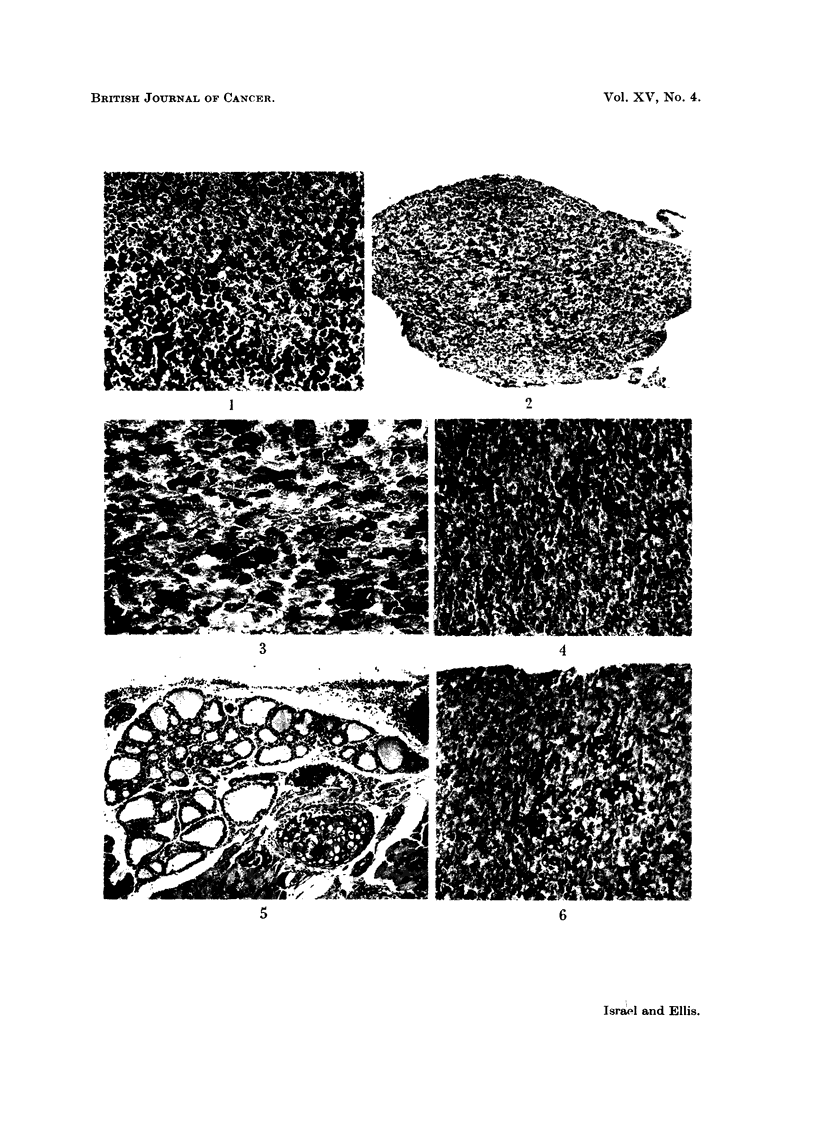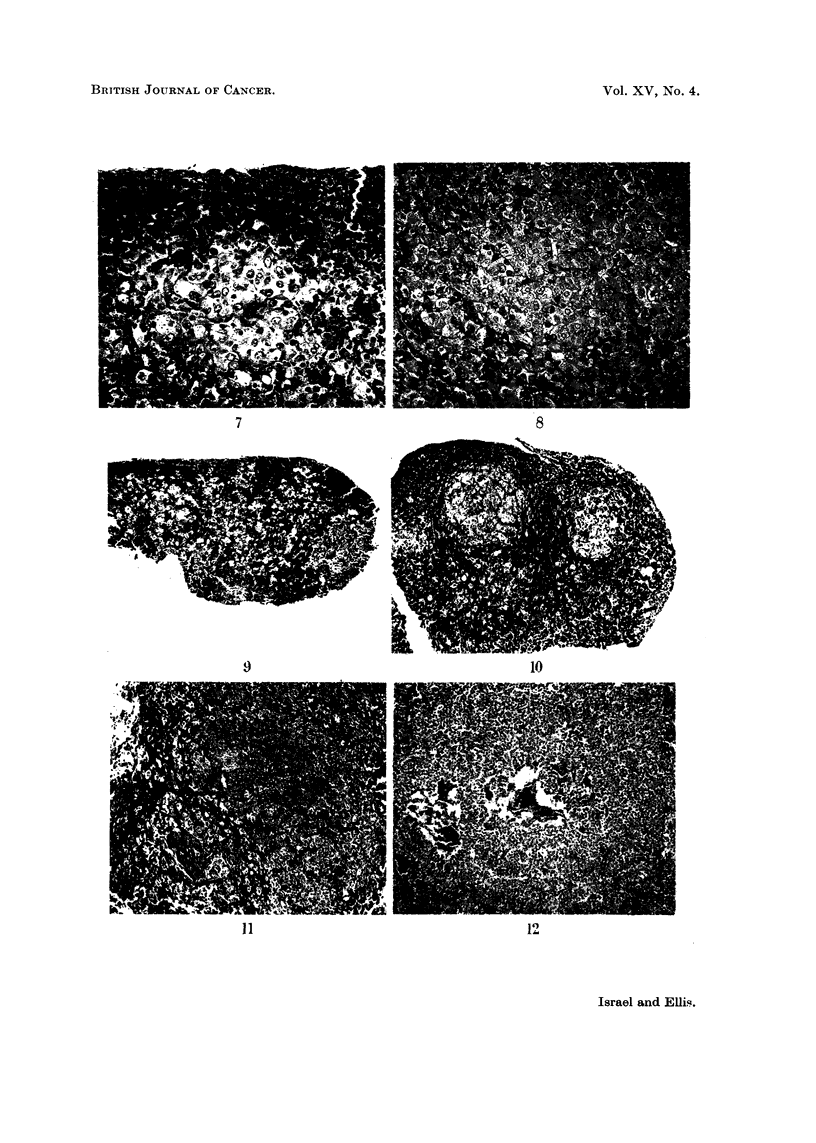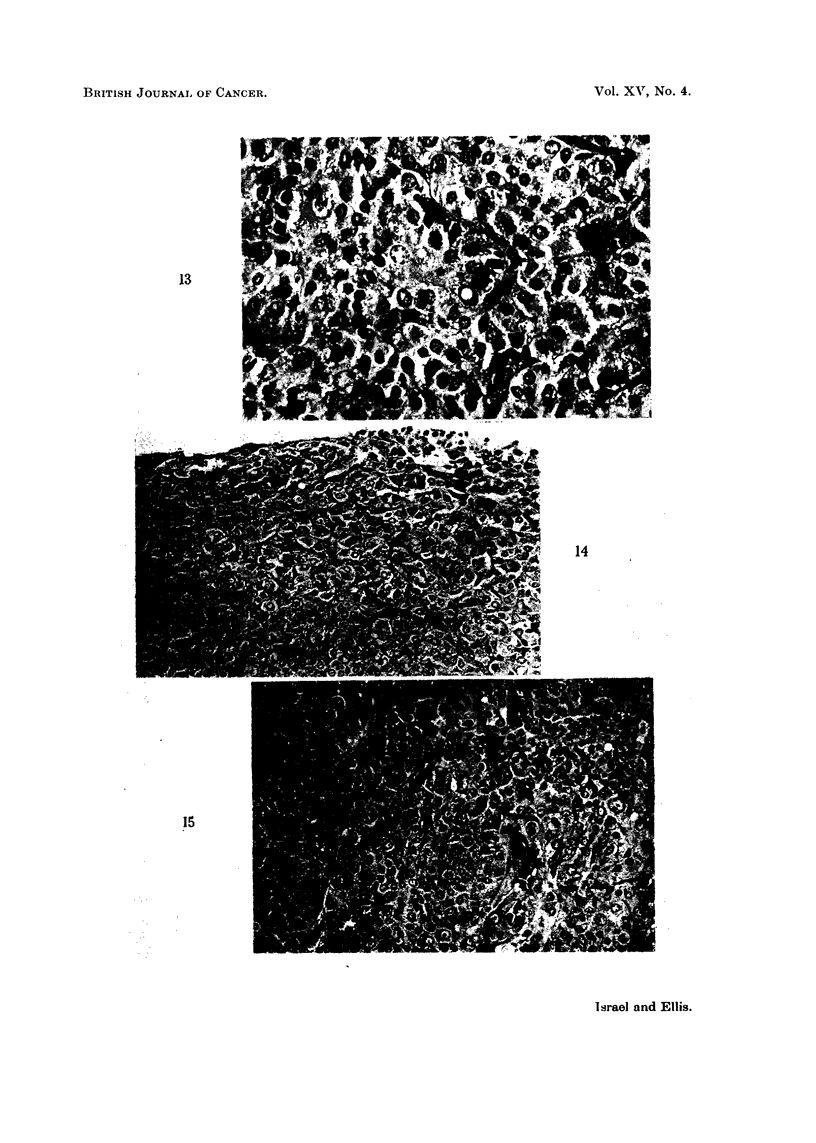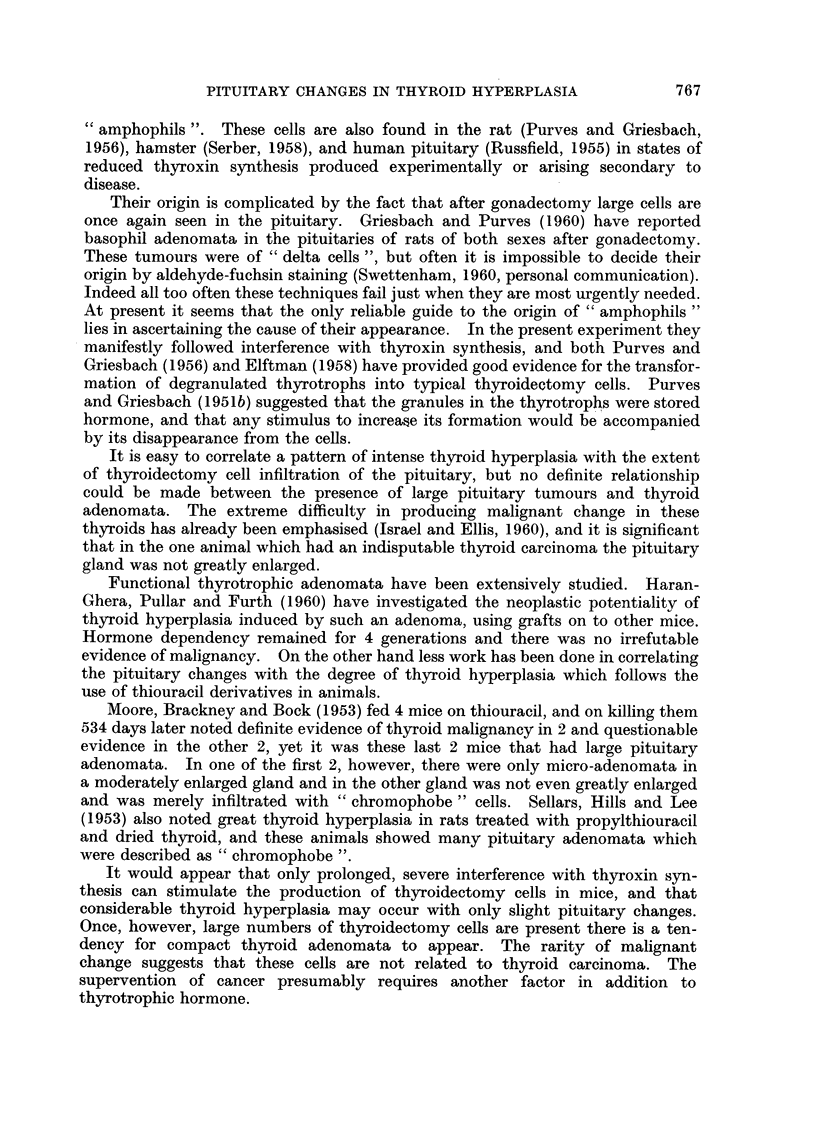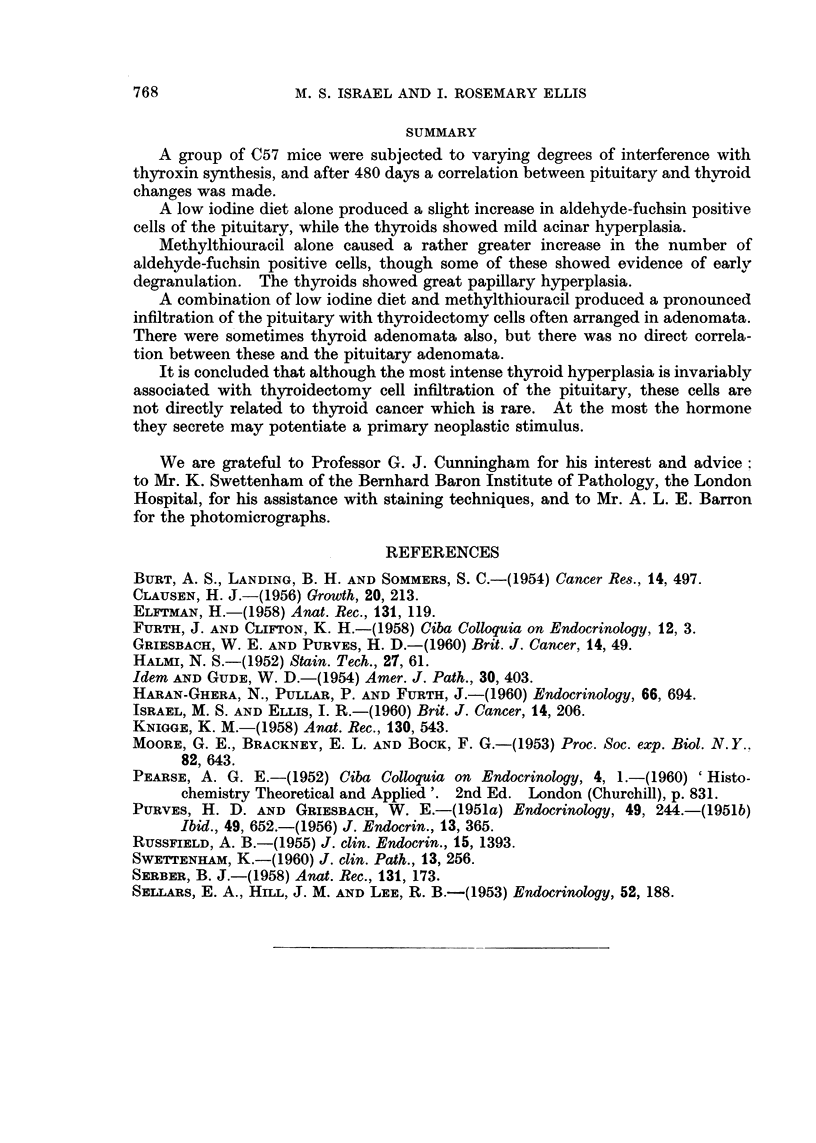# The Pituitary Changes in Mice Undergoing Thyroid Hyperplasia

**DOI:** 10.1038/bjc.1961.86

**Published:** 1961-12

**Authors:** M. S. Israel, I. Rosemary Ellis

## Abstract

**Images:**


### THE PITUITARY CHANGES IN MICE UNDERGOING THYROID HYPERPLASIA

M. S. ISRAEL AND I. ROSEMARY ELLIS

*From the Department of Pathology, Royal College of Surgeons of England, Lincoln's Inn Fields, London, W.C.2*

Received for publication October 9, 1961

It is well recognised that any condition interfering with thyroxin synthesis leads to an increased secretion of thyrotrophic hormone from the anterior pituitary. The " thyrotrophs " undergo proliferation and may form large adenomata (Furth and Clifton, 1958). The thyrotrophic hormone in turn causes thyroid hyperplasia, provided of course the original agent interfering with thyroxin formation does not actually destroy the parenchyma of the gland.

In the following experiment mice have been treated with methylthiouracil and a low iodine diet. An attempt has been made to correlate the changes in pituitary with varying degrees of hyperplasia encountered in the thyroid.

### MATERIAL AND METHODS

One hundred 4 month old C57 mice, 50 of each sex, were divided into 3 groups : a control group of 25, a group of 50 fed on methylthiouracil and a stock 41 B diet, and a group of 25 given methylthiouracil and a low iodine diet. The methylthiouracil was administered as a 0·05 per cent solution in distilled drinking water. Details of the diet have already been described (Israel and Ellis, 1960). In addition 10 mice were given the low iodine diet alone.

After 480 days the animals were killed and their thyroid glands examined, as reported previously (Israel and Ellis, 1960). The pituitary glands were removed together with a piece of underlying bone, and these were fixed either in 10 per cent formol saline or in 4 per cent formol-sublimate-saline (Swettenham, 1960). They were then dissected free from the bone, and serial paraffin sections were cut and stained as follows :

1. Erhlich's haematoxylin and eosin.
2. Mallory's trichrome stain.
3. Periodic acid Schiff (PAS) method without an orange G counterstain (Pearse, 1960).
4. Aldehyde-fuchsin stain with light green-orange G counterstain (Halmi, 1952).

### RESULTS

### A. *Control group*

The general pattern of pituitary cytology was uniform in the control group. Acidophil cells predominated in both sexes, and though basophils tended to be rather sparse, some glands showed considerable focal accumulations of these cells (Fig. 1). Chromophobes were more numerous than basophils.

The aldehyde-fuchsin stain revealed moderate numbers of angular, purple staining cells in the central areas of the lateral portions of the glands, while the surfaces showed none (Fig. 2 and 3). In the isthmic median parts of the glands, which connect the bulkier lateral portions, some green staining cells were present.

The PAS stain displayed cells on the inferior surfaces which stained varying shades of pink, and a few central angular cells were deeply positive, but this method was not helpful in distinguishing thyrotrophs from gonadotrophs.

It is interesting that while the formol mercury fixative brought out the aldehyde-fuchsin positive cells better than formol saline, it was the formol saline which gave the better results with the PAS stain, a finding previously noted by Swettenham (1960).

B. *Low iodine diet alone*

The changes were very slight ; the pituitaries were not enlarged, but there was a slight increase in the number of basophils. These stained deeply with PAS. More aldehyde-fuchsin positive cells were present centrally than in the controls (Fig. 4). The acidophils showed no change. The thyroids of these animals, not described previously, showed mild acinar hyperplasia without papillary ingrowth (Fig. 5).

C. *Methylthiouracil and stock diet*

There was a minimal degree of pituitary enlargement, which could be accounted for by a moderate increase in the number of basophils and a considerably augmented vascularity. Once again these basophils stained deep red with PAS in a diffuse manner. The coarse, dense, intensely-staining " T " granules described in rat thyrotrophs following thyroxin deficiency (Purves and Griesbach, 1956) were not encountered.

There was an increase in the number of aldehyde-fuchsin positive cells centrally, though some stained less strongly than others due to early degranulation (Fig. 6). Acidophils persisted and retained their granulation.

In some of the most hypercellular glands there were a few large, vacuolated " thyroidectomy " cells in the central zones, but they were too sparse to be prominent. The thyroids of such mice were particularly hyperplastic.

D. *Methylthiouracil and Low iodine diet together*

It required a combination of these two factors to produce really distinctive pituitary changes, and these were due to the appearance of many greatly enlarged " thyroidectomy " cells, as easily recognised by haematoxylin and eosin staining as by any more complicated techniques. They were polygonal and their faintly eosinophilic cytoplasm often had a festooned appearance due to intense vacuolation. The nuclei were large and had prominent nucleoli (Fig. 7). The trichrome and aldehyde-fuchsin methods gave negative results (Fig. 8). With the PAS stain some of the cells contained a few coarse, intensely-staining granules, somewhat reminiscent of the " T " granules of Purves and Griesbach, but most of the cells were PAS negative.

These cells were constantly present in large numbers, being especially abundant centrally (Fig. 9), and to a lesser extent peripherally. In 19 of the 25 pituitaries they were aggregated into discrete adenomata, though in 11 these were of microscopical size only. Such micro-adenomata were usually central (Fig. 10), though sometimes peripheral also (Fig. 11), and elsewhere there was a copious infiltration of similar cells.

In 8 mice the adenomata was so large that the pituitaries were macroscopically massive (Fig. 12). The cells of these adenomata were smaller, more spherical, less vacuolated and more basophilic than in the smaller collections, and they were arranged in solid clusters surrounded by a highly vascular stroma (Fig. 13). In the engorged sinusoids there were lining cells filled with haemosiderin pigment (Fig. 14), perhaps derived from previous haemorrhages in engorged glands. In some of the larger tumours mitotic figures were quite frequent (Fig. 15), but in none was there evidence of local invasion or distant metastasis. In even the largest tumours the remains of compressed parenchyma was present, and in it fully granulated acidophils were still to be found, but aldehyde-fuchsin positive cells were absent.

In all the mice there was profound thyroid hyperplasia, but there was no absolute correlation between pituitary size and thyroid proliferation. In only 4 mice with thyroid adenomata were there also pituitary adenomata, though 3 others had micro-adenomata in their pituitaries. In the remainder there was merely a diffuse infiltration of thyroidectomy cells throughout the parenchyma.

Conversely, only 4 of the 8 large pituitary adenomata were associated with thyroid adenomata.

There was no special sex incidence of pituitary adenomata.

One mouse died spontaneously of a metastasising thyroid cancer. Unfortunately the pituitary was too autolysed for adequate histological appraisal, but it did not appear to be conspicuously enlarged.

### DISCUSSION

With the development of histochemical techniques over the last decade, much progress has been made in elucidating the nature of the pituitary basophils. Pearse (1952) in particular has demonstrated the importance of the PAS stain in identifying the mucoprotein gonadotrophic and thyrothrophic hormones present in basophils and also in some chromophobes, while Halmi (1952) has employed the aldehyde-fuchsin technique to distinguish between different types of basophils. Purves and Griesbach (1951*a*) have applied these methods extensively to the rat pituitary and have been able to divide the basophils into two main groups : a central group, consisting of cells staining purple with aldehyde-fuchsin (" beta cells ") and identified as thyrotrophs, and a peripheral group, remaining unstained with aldehyde-fuchsin and taking up the light green counterstain (" delta cells "). These are believed to be gonadotrophs.

By contrast the mouse pituitary has been far less exhaustively studied. Halmi and Gude (1954) described a picture similar to that of the rat pituitary, with centrally placed aldehyde-fuchsin positive cells, and negatively staining cells near the area of the isthmus. The histological appearances of the control group described above conform to Halmi and Gude's description quite closely, though it is evident that much has still to be learnt about the cytology of the normal mouse pituitary.

The comparatively minor changes induced in the mouse pituitary by methylthiouracil alone are interesting when the great thyroid hyperplasia is remembered.

Clausen (1956) fed rats on thiouracil and also noted moderate basophil hyperplasia after one year, most of the cells being aldehyde-fuchsin positive. By 18 months many of the basophils were degranuated, yet thyroidectomy cells were very scanty. Only after 2 years were small micro-adenomata to be found. Thyroxin deficiency in rats, whether due to thyroidectomy (Knigge, 1958) or following thiouracil administration (Sellars, Hill and Lee, 1953), produces a rapid degranulation of acidophils. This does not occur in mice (Halmi and Gude, 1954), a feature the mouse pituitary has in common with that of the hamster (Serber, 1958).

It required a combination of low iodine diet and methylthiouracil to produce the very conspicuous " thyroidectomy cell " in large numbers, sometimes to the extent of forming large tumours almost completely obliterating the normal parenchyma. It is noteworthy that these cells are recognised quite as easily by haematoxylin and eosin staining as by any histochemical methods, as they are PAS and aldehyde-fuchsin negative.

The nature of these cells has long given rise to speculation because of their indeterminate staining with trichrome methods and their obvious morphological dissimilarity to the common chromophobe. Halmi and Gude (1954) produced adenomata composed of these cells by subjecting mice to radiothyroidectomy, and Burt, Landing and Sommers (1954) administered radioactive iodine to the same effect. These latter authors described the cells in great detail, and noting their ambivalent staining reactions with commonly used techniques, called them

---

### EXPLANATION OF PLATES

FIG. 1.—Normal mouse pituitary. Both acidophils and basophils are shown, but the basophils stain much more darkly, and are rather numerous in this field. Mallory's trichrome. ×165.

FIG. 2.—Normal mouse pituitary. A lateral portion of the anterior lobe showing the presence of darkly-staining cells in the central area only. Halmi aldehyde-fuschin. ×75.

FIG. 3.—Normal mouse pituitary. A detailed view of the central area of Fig. 2 showing the arrangement of positively-staining cells. Halmi aldehyde-fuchsin. ×300.

FIG. 4.—Pituitary of mouse fed on low iodine diet. Some increase in number of positively-staining cells in the central part. Halmi aldehyde-fuchsin. ×165.

FIG. 5.—Thyroid of mouse fed on low iodine diet. There is mild acinar hyperplasia only. Haematoxylin and eosin. ×112.

FIG. 6.—Pituitary of mouse given methylthiouracil. An increased number of central positively-staining cells, some of which show evidence of early degranulation. Halmi aldehyde-fuchsin. ×165.

FIG. 7.—Pituitary of mouse fed on methylthiouracil and low iodine diet. Typical " thyroidectomy " cells in a central cluster. Haematoxylin and eosin. ×165.

FIG. 8.—Pituitary of mouse fed on methylthiouracil and low iodine diet. The thyroidectomy cells do not stain with aldehyde-fuchsin, but a few remaining small cells stain positively. Halmi aldehyde-fuchsin. ×165.

FIG. 9.—A diffuse central infiltration of thyroidectomy cells and a small micro-adenoma peripherally. Mallory's trichrome. ×82.

FIG. 10.—Two central micro-adenomata. Mallory's trichrome. ×82.

FIG. 11.—A large peripheral microadenoma. The vacuolation of the cells is evident. Mallory's trichrome. ×165.

FIG. 12.—A large adenoma. Haematoxylin and eosin. ×82.

FIG. 13.—The arrangement of adenoma cells in solid clumps is seen. The cells are quite vacuolated. A mitotic figure is present. Haematoxylin and eosin. ×410.

FIG. 14.—Many of the sinus-lining cells of the adenoma contain iron pigment. Perls's stain with neutral red counterstain. ×205.

FIG. 15.—An exuberant adenoma with mitotic figures. Haematoxylin and eosin. ×205.

1

2

3

4

5

6

7

8

9

10

11

12

13

14

15

" amphophils ". These cells are also found in the rat (Purves and Griesbach, 1956), hamster (Serber, 1958), and human pituitary (Russfield, 1955) in states of reduced thyroxin synthesis produced experimentally or arising secondary to disease.

Their origin is complicated by the fact that after gonadectomy large cells are once again seen in the pituitary. Griesbach and Purves (1960) have reported basophil adenomata in the pituitaries of rats of both sexes after gonadectomy. These tumours were of " delta cells ", but often it is impossible to decide their origin by aldehyde-fuchsin staining (Swettenham, 1960, personal communication). Indeed all too often these techniques fail just when they are most urgently needed. At present it seems that the only reliable guide to the origin of " amphophils " lies in ascertaining the cause of their appearance. In the present experiment they manifestly followed interference with thyroxin synthesis, and both Purves and Griesbach (1956) and Elftman (1958) have provided good evidence for the transformation of degranulated thyrotrophs into typical thyroidectomy cells. Purves and Griesbach (1951*b*) suggested that the granules in the thyrotrophs were stored hormone, and that any stimulus to increase its formation would be accompanied by its disappearance from the cells.

It is easy to correlate a pattern of intense thyroid hyperplasia with the extent of thyroidectomy cell infiltration of the pituitary, but no definite relationship could be made between the presence of large pituitary tumours and thyroid adenomata. The extreme difficulty in producing malignant change in these thyroids has already been emphasised (Israel and Ellis, 1960), and it is significant that in the one animal which had an indisputable thyroid carcinoma the pituitary gland was not greatly enlarged.

Functional thyrotrophic adenomata have been extensively studied. Haran-Ghera, Pullar and Furth (1960) have investigated the neoplastic potentiality of thyroid hyperplasia induced by such an adenoma, using grafts on to other mice. Hormone dependency remained for 4 generations and there was no irrefutable evidence of malignancy. On the other hand less work has been done in correlating the pituitary changes with the degree of thyroid hyperplasia which follows the use of thiouracil derivatives in animals.

Moore, Brackney and Bock (1953) fed 4 mice on thiouracil, and on killing them 534 days later noted definite evidence of thyroid malignancy in 2 and questionable evidence in the other 2, yet it was these last 2 mice that had large pituitary adenomata. In one of the first 2, however, there were only micro-adenomata in a moderately enlarged gland and in the other gland was not even greatly enlarged and was merely infiltrated with " chromophobe " cells. Sellars, Hills and Lee (1953) also noted great thyroid hyperplasia in rats treated with propylthiouracil and dried thyroid, and these animals showed many pituitary adenomata which were described as " chromophobe ".

It would appear that only prolonged, severe interference with thyroxin synthesis can stimulate the production of thyroidectomy cells in mice, and that considerable thyroid hyperplasia may occur with only slight pituitary changes. Once, however, large numbers of thyroidectomy cells are present there is a tendency for compact thyroid adenomata to appear. The rarity of malignant change suggests that these cells are not related to thyroid carcinoma. The supervention of cancer presumably requires another factor in addition to thyrotrophic hormone.

SUMMARY

A group of C57 mice were subjected to varying degrees of interference with thyroxin synthesis, and after 480 days a correlation between pituitary and thyroid changes was made.

A low iodine diet alone produced a slight increase in aldehyde-fuchsin positive cells of the pituitary, while the thyroids showed mild acinar hyperplasia.

Methylthiouracil alone caused a rather greater increase in the number of aldehyde-fuchsin positive cells, though some of these showed evidence of early degranulation. The thyroids showed great papillary hyperplasia.

A combination of low iodine diet and methylthiouracil produced a pronounced infiltration of the pituitary with thyroidectomy cells often arranged in adenomata. There were sometimes thyroid adenomata also, but there was no direct correlation between these and the pituitary adenomata.

It is concluded that although the most intense thyroid hyperplasia is invariably associated with thyroidectomy cell infiltration of the pituitary, these cells are not directly related to thyroid cancer which is rare. At the most the hormone they secrete may potentiate a primary neoplastic stimulus.

We are grateful to Professor G. J. Cunningham for his interest and advice ; to Mr. K. Swettenham of the Bernhard Baron Institute of Pathology, the London Hospital, for his assistance with staining techniques, and to Mr. A. L. E. Barron for the photomicrographs.

### REFERENCES

BURT, A. S., LANDING, B. H. AND SOMMERS, S. C.—(1954) *Cancer Res.*, **14**, 497.
CLAUSEN, H. J.—(1956) *Growth*, **20**, 213.
ELFTMAN, H.—(1958) *Anat. Rec.*, **131**, 119.
FURTH, J. AND CLIFTON, K. H.—(1958) *Ciba Colloquia on Endocrinology*, **12**, 3.
GRIESBACH, W. E. AND PURVES, H. D.—(1960) *Brit. J. Cancer*, **14**, 49.
HALMI, N. S.—(1952) *Stain. Tech.*, **27**, 61.
*Idem* AND GUDE, W. D.—(1954) *Amer. J. Path.*, **30**, 403.
HARAN-GHERA, N., PULLAR, P. AND FURTH, J.—(1960) *Endocrinology*, **66**, 694.
ISRAEL, M. S. AND ELLIS, I. R.—(1960) *Brit. J. Cancer*, **14**, 206.
KNIGGE, K. M.—(1958) *Anat. Rec.*, **130**, 543.
MOORE, G. E., BRACKNEY, E. L. AND BOCK, F. G.—(1953) *Proc. Soc. exp. Biol. N.Y.*, **82**, 643.
PEARSE, A. G. E.—(1952) *Ciba Colloquia on Endocrinology*, **4**, 1.—(1960) ‘Histochemistry Theoretical and Applied’. 2nd Ed. London (Churchill), p. 831.
PURVES, H. D. AND GRIESBACH, W. E.—(1951*a*) *Endocrinology*, **49**, 244.—(1951*b*) *Ibid.*, **49**, 652.—(1956) *J. Endocrin.*, **13**, 365.
RUSSFIELD, A. B.—(1955) *J. clin. Endocrin.*, **15**, 1393.
SWETTENHAM, K.—(1960) *J. clin. Path.*, **13**, 256.
SERBER, B. J.—(1958) *Anat. Rec.*, **131**, 173.
SELLARS, E. A., HILL, J. M. AND LEE, R. B.—(1953) *Endocrinology*, **52**, 188.